# Synergistic Antibacterial Mechanism of Benzyl Isothiocyanate and Resveratrol Against *Staphylococcus aureus* Revealed by Transcriptomic Analysis and Their Application in Beef

**DOI:** 10.3390/foods14091610

**Published:** 2025-05-01

**Authors:** Jianan Liu, Jinle Ma, Yingrui Wang, Hongshun Hao, Jingran Bi, Hongman Hou, Gongliang Zhang

**Affiliations:** 1 School of Food Science and Technology, Dalian Polytechnic University, No. 1, Qinggongyuan, Ganjingzi District, Dalian 116034, China; liujianan_xg@163.com (J.L.); 18340658166@163.com (J.M.); yrw0831@163.com (Y.W.); bijingran1225@foxmail.com (J.B.); houhongman2011@hotmail.com (H.H.); 2 Liaoning Key Lab for Aquatic Processing Quality and Safety, Dalian Polytechnic University, No. 1, Qinggongyuan, Ganjingzi District, Dalian 116034, China; 3 Department of Inorganic Nonmetallic Materials Engineering, Dalian Polytechnic University, No. 1, Qinggongyuan, Ganjingzi District, Dalian 116034, China; beike1952@163.com

**Keywords:** synergistic antibacterial mechanism, *Staphylococcus aureus*, benzyl isothiocyanate, resveratrol, transcriptome

## Abstract

This study aims to elucidate the synergistic antibacterial mechanism of benzyl isothiocyanate (BITC) and resveratrol (RES) on *Staphylococcus aureus* (*S. aureus*) at the transcriptional level. Compared with the individuals, the combination of BITC and RES (BITC_RES) reduced *S. aureus* growth, inhibited biofilm formation, and increased cell membrane disruption. The transcriptomic results showed that the BITC_RES group presented 245 and 1150 more DEGs than the BITC group and the RES group, respectively. In addition, some other key genes in the BITC_RES group, including serine protease (*splA*, *splE*), Sae regulatory system (*saeR*, *saeS*, *tsaE*, *sau300*), accessory gene regulator protein C (*agrC*), cysteine protease (*sspB*), glutamyl endopeptidase (*sspA*), and hemolysin toxin family-related genes (*hly*, *lukDv*, *lukEv*), and the relative expression of these 12 genes was downregulated by 2.2–259.8-fold, 0.8–259.8-fold and 1.2–158.2-fold greater than those in the BITC group and the RES group, respectively. Finally, a synergistic antimicrobial effect of this combination was also observed in fresh lean beef at 4 °C and 25 °C. These findings provide information for future studies on the synergistic antimicrobial effects of BITC and RES on *S. aureus*.

## 1. Introduction

*Staphylococcus aureus* (*S. aureus*), which is a foodborne pathogen, can infect humans and animals in different ways [1]. It produces enterotoxins that can lead to food poisoning and even endocarditis, sepsis, bacteremia, vertebral osteomyelitis, and abscess infections [2]. Currently, bacterial infections are largely treated by antibiotics, such as tetracycline and chloramphenicol [3]. However, the indiscriminate use of antibiotics can cause bacteria to develop resistance. Therefore, it is necessary to find a natural product to inhibit bacterial infections.

Natural products are the most commonly used antimicrobial agents. Benzyl isothiocyanate (BITC) is an organosulfide, widely used as an antimicrobial agent [4]. For example, several studies have reported that BITC had significant antibacterial effects on *Escherichia coli* (*E. coli*) O157:H7, *Salmonella typhimurium* (*S. typhimurium*), and *S. aureus*, including the inhibition of biofilm formation, suppression in the expression of some genes, and disruption of cellular integrity [5,6,7,8]. Resveratrol (RES) is a polyphenol compound found in peanuts, edibles, grapes, and mulberries that is colorless and odorless at low concentrations, and has good antibacterial properties [9]. RES had good antibacterial effects on *S. aureus* and *Salmonella typhimurium* ATCC 13311, including the suppression of biofilm formation, reduction in motility, and disruption of colony sensing [9,10]. Studying synergistic bacterial inhibition is a good strategy. Synergism means that a combination of low concentrations of antimicrobials can achieve the level of action of a high concentration of antimicrobials alone [11]. Since BITC has a certain flavor, the concentration of BITC is reduced in the synergistic combination so that the taste of BITC becomes mild, thus having no negative effects on the organoleptic qualities, as well as achieving consumer acceptance. Therefore, the combination of antimicrobial agents may reduce the concentration of a single antimicrobial agent, resulting in better antimicrobial activity. Synergistic antimicrobial applications of various antimicrobial agents have been extensively studied. For example, the combination of phenethyl isothiocyanate and auranofin was more effective at preventing *S. aureus* infection than either agent alone [12]. Sucrose laurate also had synergistic antimicrobial effects with nisin on *S. aureus* [13]. Moreover, the synergistic antimicrobial effects of BITC and RES on *Listeria monocytogenes* (*L. monocytogenes*), including effects on ROS levels, cell morphology, biofilm formation, the cell membrane, and cell motility, have been reported [14]. However, the synergistic antimicrobial mechanism of BITC and RES against *S. aureus* has not been reported.

Transcriptome sequencing (RNA-Seq), a tool that allows for the quantification of gene expression via the sequencing of mRNA transcripts, has been widely applied in bacteria [15]. Das et al. explored the synergistic effects of ceftriaxone and cranberry pomace extracts at subinhibitory concentrations on extracranial *E. coli* via RNA-seq [16]. Furthermore, studies have analyzed the synergistic antibacterial activities of p-anisaldehyde and epigallocatechin gallate against *Pseudomonas aeruginosa* (*P. aeruginosa*), as well as cellular pathways via transcriptomics [17]. A polymyxin–rifampicin cotreatment of *P. aeruginosa* increased the peptidoglycan biosynthesis gene expression [18]. However, the synergistic mechanism of BITC and RES against *S. aureus* has not been reported at the transcriptional level.

In addition, synergistic effects have been widely used in meat. For example, the cell wall hydrolase Lys14579 and cinnamaldehyde showed synergistic antimicrobial activity against *Bacillus cereus* on inoculated lean beef [19]. Endolysin LysSA97, in combination with carvacrol, also exhibited synergistic effects on *S. aureus* inoculated on beef [20]. However, studies on the synergistic antimicrobial effects of BITC and RES on *S. aureus* inoculated on lean beef have not been reported.

This study aims to elucidate the synergistic antibacterial mechanism of BITC and RES against *S. aureus*. The synergistic antimicrobial effects of BITC and RES against *S. aureus* were verified by biofilm formation, cell morphology, cell membrane permeability, and transcriptomics. Finally, the inhibitory effect of BITC and RES on beef inoculated with *S. aureus* at different temperatures was investigated. The synergistic effects of BITC and RES provide useful information for the development of novel food preservatives.

## 2. Materials and Methods

### 2.1. Bacterial Strains and Chemicals

Benzyl isothiocyanate (BITC) was provided by Sigma (Sigma-Aldrich, MA, USA). Resveratrol (RES), eugenol, and carvacrol were obtained from Aladdin (Aladdin Reagent Co., Ltd., Shanghai, China). The Food Microbiology Laboratory of Dalian University of Technology (China) provided *S. aureus* ATCC 6538.

### 2.2. Fractional Inhibitory Concentration Index (FICI)

The MICs of BITC and RES for *S. aureus* were assessed via the broth microdilution technique [21]. No bacterial growth was detected during incubation, and this concentration was considered the MIC. The synergistic antimicrobial effects of BITC and RES on *S. aureus* were evaluated via the checkerboard methods [22]. In brief, two different antibacterial agents were sequentially diluted to concentrations ranging from 1/16 MIC to 2 MIC in the transverse and longitudinal directions of a 96-well plate. The FICI was used to assess the synergistic antimicrobial effects of each combination and was calculated as follows:

(1) FICI=FICBITC+FICRES=MICBITC in combination/MICBITC alone+MICRES in combination/MICRES alone

The FICI also has more than 1 interpretation, including synergistic (FICI ≤ 0.5), additivity (0.5 < FICI < 1), indifferent (1 ≤ FICI ≤ 4), and antagonism (FICI > 4).

### 2.3. Time-Dependent Killing Curve

A suspension of 10^6^ CFU/mL of *S. aureus* bacteria was treated with 1/4 MIC BITC (BITC), 1/4 MIC RES (RES), or 1/8 MIC BITC + 1/8 MIC RES (BITC_RES) at 37 °C. The suspensions were taken at 0, 4, 8, 12, 16, and 24 h, and the number of surviving bacteria was counted with tryptone soy agar medium plates.

### 2.4. Antibiofilm Formation

Approximately 10^6^ CFU/mL of *S. aureus* suspension was placed in a 96-well plate with BITC, RES, and BITC_RES, and was incubated at 37 °C for 48 h. The control samples did not contain BITC or RES. Biofilm formation was followed by removal of floating cells with sterile PBS and then fixation of the biofilm with methanol for 15 min. The preserved biofilms were dyed with 1% crystal violet solution for 10 min. Finally, 33% glacial acetic acid (200 µL) was added to each well. At last, the absorbance was recorded at 590 nm with a microplate reader (SpectraMax M2, SpectraMax, La Jolla, CA, USA).

### 2.5. Scanning Electron Microscopy (SEM)

The bacterial morphology was observed via SEM. A bacterial suspension was added on a coverslip in a 6-well polystyrene plate with BITC, RES, and BITC_RES, and incubated at 37 °C for 48 h. The SEM samples were prepared according to previous studies [23]. The samples were visualized via field emission SEM (Quanta 450, Waltham, MA, USA).

### 2.6. Cell Membrane Analysis

#### 2.6.1. Integrity of the Cell Membrane

The integrity of the cell membranes was monitored via SYTO 9^TM^ and propidium iodide (PI) [24]. *S. aureus* suspensions were cocultured with BITC, RES, or BITC_RES for 8 h at 37 °C. The control consisted of a sample without BITC or RES. The cells in the treatment and control groups were stained with SYTO 9^TM^ (KeyGEN BioTECH, Nanjing, China) and PI (Solarbio, Beijing, China) for 20 min. Lastly, the fluorescence images were obtained via fluorescence microscopy (Nikon, Eclipse Ti, Tokyo, Japan).

#### 2.6.2. Nucleic Acid and Protein Concentrations

The macromolecular release of bacteria was determined according to the methods of Ma et al. [24]. *S. aureus* suspensions were cocultured with BITC, RES, or BITC_RES at 37 °C. The control consisted of a sample without BITC or RES. Finally, the amount of nucleic acid leakage at 260 nm was measured by a microplate reader after 8 h. Moreover, the amount of protein released was subsequently determined after 8 h via a bicinchoninic acid assay (Solarbio, Beijing, China).

#### 2.6.3. Intimal Permeability

Determination of the intimal permeability of *S. aureus* was determined via measurement of β-galactosidase activity [23]. *S. aureus* suspensions were co-cultured with BITC, RES, or BITC_RES for 8 h at 37 °C. The control consisted of a sample without BITC or RES. An amount of 50 mM ONPG and 0.5 mol/L Na_2_CO_3_ were used for the sample reaction and termination. The β-galactosidase activity released by *S. aureus* was measured at 420 nm via a microplate reader. The formula is as follows:

(2) β−Galactosidase activity (U/mL)=OD420 nm×2.778

#### 2.6.4. Plasma Membrane Potential

Ning’s method was used with slight modifications [25]. *S. aureus* suspensions were cocultured with BITC, RES, or BITC_RES for 8 h at 37 °C. The control consisted of a sample without BITC or RES, and 5 μM rhodamine 123 (Solarbio, Beijing, China) was added to the *S. aureus* suspension. The treated *S. aureus* suspension was washed in PBS. The membrane potential was determined by measuring the fluorescence intensity with a microplate reader. Rhodamine 123 was excited and emitted at 488 nm and 530 nm, respectively.

### 2.7. Synergistic Effects of BITC and RES on S. aureus at the Transcriptional Level

#### 2.7.1. Sample Preparation

A total of 10^6^ CFU/mL bacterial broth was coincubated with 1/8 MIC BITC (BITC), 1/8 MIC RES (RES), or 1/8 MIC BITC + 1/8 MIC RES (BITC_RES) at 37 °C for 12 h. Three parallel sets were set up for all groups.

#### 2.7.2. Library Construction and Sequencing

The library construction process was carried out in several ways. Total RNA sample identification, mRNA enrichment, duplex cDNA synthesis, end repair, A-addition and splicing, fragment selection and PCR amplification, library construction, and Illumina sequencing were performed.

#### 2.7.3. Bioinformatic Analysis

The raw sequence data were obtained via throughput sequencing, and low-quality reads were removed to obtain clean reads for final use. The filtered sequences were analyzed for genomic localization via Bowtie2 (version 2.5.2) software. The differentially expressed genes (DEGs) obtained after screening were imported into the Gene Ontology (GO) comprehensive database and the Kyoto Encyclopedia of Genes and Genomes (KEGG) comprehensive database to find the corresponding functional annotations and metabolic pathways for enrichment analysis.

### 2.8. Quantitative Real-Time Polymerase Chain Reaction (qRT-PCR)

*S. aureus* was coincubated with BITC, RES, or BITC_RES for 12 h, after which total RNA was extracted. A qRT-PCR analysis was performed using SYBR^®^ Advanced^TM^ II (Takara, Beijing, China). Thirteen genes in *S. aureus*, including serine protease SplD (*splD*), serine protease SplA (*splA*), serine protease SplE (*splE*), response regulator (*saeR*), sensor histidine kinase SaeS (*saeS*), threonylcarbamoyladenosine biosynthesis protein (tsaE), type VII secretion system protein (*sau300*), accessory gene regulator protein C (*agrC*), cysteine protease (*sspB*), glutamyl endopeptidase (*sspA*), alpha-hemolysin (*hly*), leukotoxin LukD (*lukDv*) and leukotoxin LukE (*lukEv*), were studied. Table S1 lists the specific primers designed for the differential gene screening of the RNA sequences via Primer 5.0 software. The DEG levels were assessed via the 2^−ΔΔCt^ method, where the endogenous gene used was the 16S rRNA gene [26].

### 2.9. Synergistic Effect of BITC and RES on S. aureus on Lean Beef

Beef was cut into pieces (2 × 2 × 2 cm) and inoculated with *S. aureus* to achieve a final concentration of *S. aureus* on beef of 10^4^ CFU/g. Then BITC (1/4 MIC), RES (1/4 MIC), and BITC_RES (1/4 MIC BITC+1/4 MIC RES) were applied to the surface of the beef, and the samples were stored at 4 °C and 25 °C for 12 days. Samples were taken on 0, 3, 6, 9, and 12 d to assess the number of *S. aureus* in the beef using a selective medium for *S. aureus*.

### 2.10. Statistical Analysis

All results are presented as a mean ± standard deviation. Data were processed by SPSS (version 17.0). Differences between groups were analyzed by one-way ANOVA followed by the LSD post hoc test, with statistical significance set at *p* < 0.05.

## 3. Results

### 3.1. Synergistic Effects of BITC and RES on the Growth Inhibition of S. aureus

**Figure 1:**
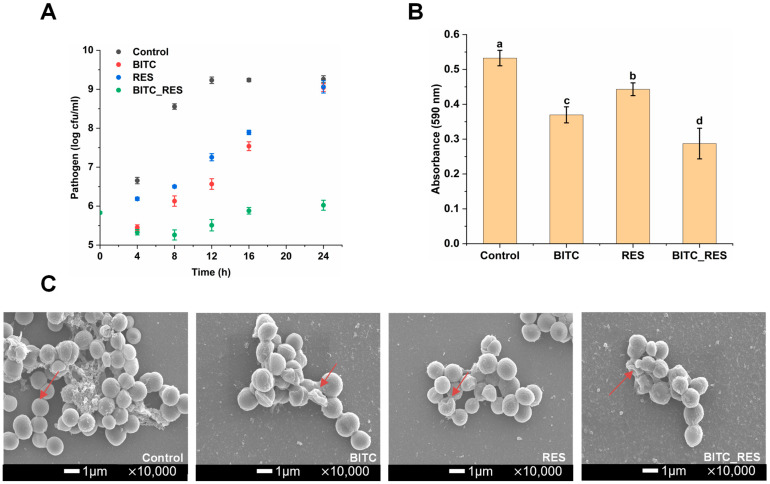
Synergistic effects of benzyl isothiocyanate (BITC) and resveratrol (RES) on the growth and ROS production of *S. aureus*: (**A**) timed killing kinetic curves; (**B**) biofilm formation; (**C**) cell morphology. BITC: 1/4 MIC BITC; RES: 1/4 MIC RES; BITC_RES: 1/8 MIC BITC+1/8 MIC RES. Different letters represent significant differences.

The FICI is the most commonly used test for synergistic antimicrobial effects between substances [27]. In our preliminary study, we selected three polyphenols and BITC for their synergistic antimicrobial effects against *S. aureus*. The MICs of BITC, RES, carvacrol, and eugenol against *S. aureus* were 0.0746, 0.2, 0.5, and 5 mg/mL, respectively. The interactions of BITC with RES, carvacrol, and eugenol were demonstrated with a checkerboard. The combination of BITC with RES, carvacrol, or eugenol had synergistic effects on *S. aureus*. Most importantly, BITC showed the best synergism with RES, with a FICI of 0.375. The study also revealed that nisin and allyl isothiocyanate synergistically inhibited *S. aureus* and *S. typhimurium*, with FICIs of less than 0.5 [28]. The combination of alpha-mangostin and RES had a FICI value of 0.094 against *S. aureus*, indicating good synergy [29]. The time-killing kinetic curves of BITC, RES, and BITC_RES against *S. aureus* after 24 h are shown in Figure 1A. Relative to the negative control, BITC resulted in a decrease in the *S. aureus* concentration within 4 h. However, the antimicrobial activity of BITC gradually decreased after 4 h. RES alone also possessed antimicrobial activity, which diminished with time. Unsurprisingly, the synergistic effect of BITC and RES resulted in no increase in the concentration of *S. aureus* over 24 h. Compared with the individual groups, the BITC_RES group presented a 3.0 log CFU/mL lower colony count of *S. aureus* at 24 h. Qi et al. reported that RES in combination with polymyxin B reduced *P. aeruginosa* by about 2.0 log CFU/mL compared with the individual groups [30]. In addition, BITC_RES reduced *L. monocytogenes* by 2.6 log CFU/mL compared to BITC or RES alone [14]. These results demonstrate that there is synergy between BITC and RES against *S. aureus*.

### 3.2. Synergistic Effects of BITC and RES on S. aureus Biofilm Formation

The results of biofilm formation by BITC-, RES-, and BITC_RES-treated *S. aureus* are shown in Figure 1B. The biofilm formation in the BITC group and the RES group was reduced by 30.6% and 16.8%, respectively, compared with those in the control group (*p* < 0.05). However, the BITC_RES group more significantly reduced biofilm formation by 46.1%. Therefore, the best inhibitory effect was clearly observed in the BITC_RES group. Chen et al. reported that phenethyl isothiocyanate could not inhibit biofilm formation, and the biofilm inhibition rate of *S. aureus* treated with auranofin at 2 × MIC was 75.6%, whereas the combination with phenethyl isothiocyanate at the MIC resulted in an inhibition rate of 91.5%, indicating that the combination had synergistic effects on the biofilm formation of *S. aureus* compared with auranofin alone [31]. The cell morphology and cell membrane integrity can be visualized via SEM. The bacterial cells in the control group were spherical, smooth, and intact. Most of the *S. aureus* cells treated with BITC no longer had an intact morphology, indicating that BITC disrupted the cell membrane (Figure 1C). Figure 1C shows that RES was not sufficiently disruptive to the cell membrane. BITC_RES was strongly disruptive to the cell membrane, which was attributed to the presence of BITC. In addition, nisin could enhance the disruptive effect of carvacrol on *S. aureus* cell membranes, demonstrating a significant synergistic antibacterial effect of nisin and carvacrol in disrupting cell membrane integrity [21]. Therefore, these results suggest that the inhibition of biofilm formation may be important for the synergistic inhibition of BITC and RES on *S. aureus*.

### 3.3. Synergistic Effects of BITC and RES on the Cell Membrane Permeability of S. aureus

**Figure 2:**
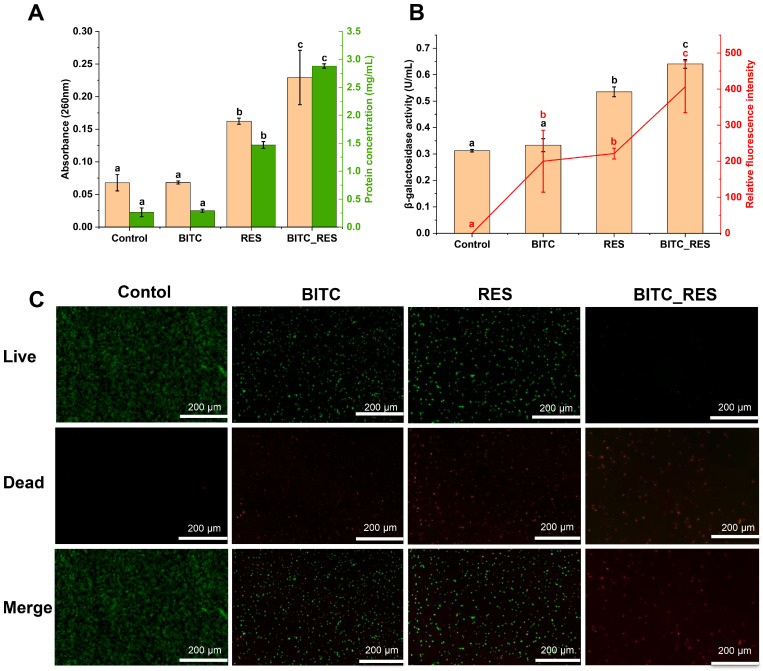
Synergistic effects of BITC and RES on the cell membrane of *S. aureus*: (**A**) leaked nucleic acids and leaked protein concentrations; (**B**) inner membrane permeability and membrane potential; (**C**): fluorescence images; BITC: 1/4 MIC BITC; RES: 1/4 MIC RES; BITC_RES: 1/8 MIC BITC + 1/8 MIC RES. Different letters represent significant differences.

Nucleic acids and proteins are found in the cytoplasm of the cell, and are biomolecules necessary for life activities [32]. The cells were co-incubated with BITC, RES, or BITC_RES for 4 h, the absorbance of the BITC_RES group at 260 nm was 0.2293, which was 39.6% and 95.1% greater than that of the BITC group and RES group, respectively (Figure 2A). The absorbances at 280 nm were 0.2928 and 1.4691 in the BITC group and RES group, respectively, and those in the BITC_RES group were 9.6 times and 2.0 times greater than those in the individual groups, respectively (Figure 2A). These results suggest that BITC_RES synergistically disrupts cell membranes. Therefore, it is demonstrated that BITC_RES plays a key role in disrupting the cell membrane of *S. aureus*. It has also been shown that single sucrose laurate and nisin treatment caused slight leakage of nucleic acids and proteins from *S. aureus*, whereas the combination of sucrose laurate and nisin treatment resulted in significant nucleic acid and protein leakage [13].

β-galactosidase is a naturally occurring enzyme in the cell cytoplasm [33]. Once the bacterial inner membrane is penetrated, cytoplasmic β-galactosidase leaks into the external environment. Therefore, β-galactosidase leakage was measured to assess the effects of BITC and RES in combination on the permeability of *S. aureus* endosomes. The leakage of β-galactosidase in the BITC group and RES group were 0.3333 and 0.5350, respectively (Figure 2B). However, the BITC_RES group had 93.6% and 32.2%, greater percentages than the BITC group and RES group, suggesting that BITC_RES was better at disrupting endomembrane permeability. Similar studies have shown that cotreatment of Lysqdvp001 with ε-poly-lysine resulted in increasing leakage of β-galactosidase, which was key to improving antibacterial efficiency through membrane damage, more significantly than for the individual groups [25]. In addition, carvacrol exacerbated membrane disruption by monocaprin, in a massive leakage of β-galactosidase from *E. coli* [24]. The membrane potential is an essential index of cell viability and metabolism [34]. As the cell membrane depolarizes, the fluorescence signal increases [35]. Depolarization of the membrane can cause a loss of permeability and affect cell growth through the inhibition of nutrient transport [36]. Figure 2B shows that the relative fluorescence values after BITC, RES, and BITC_RES treatments were 199.8, 221.2, and 406.4, respectively, suggesting that membrane depolarization appeared and BITC and RES had synergistic effects on membrane potential dissipation. The treatment of *S. aureus* with sucrose laurate in combination with nisin also depolarized the membrane potential and exerted a synergistic effect [13]. The synergistic inhibition mechanism of BITC and RES was further analyzed using SYTO 9^TM^ and PI for a cell membrane integrity assay of *S. aureus*. The cells without the disrupted cell membranes showed strong green, fluorescent signals, and cells in the BITC group or the RES group also showed more green, fluorescent signals, which indicated that the permeability of the cell membranes did not undergo much change in the individual treatment groups (Figure 2C). In addition, intense red fluorescence was triggered by treatment with BITC_RES, suggesting that BITC and RES can synergistically disrupt cell membrane integrity and even cause cell death. It has also been shown that a combination treatment of *S. aureus* with DNase I and lysostaphin caused greater disruption of the cell membrane [37]. In addition, sucralose laurate and nisin in combination increased the permeability of the cell membrane, thereby increasing the antibacterial efficiency of nisin against *S. aureus* [13]. Therefore, these results illustrate that BITC_RES better disrupts the cell membrane integrity, suggesting a greater synergistic role of BITC and RES in the cell membrane permeability of *S. aureus*. 

### 3.4. Synergistic Effects of BITC and RES on the DEGs of S. aureus

**Figure 3:**
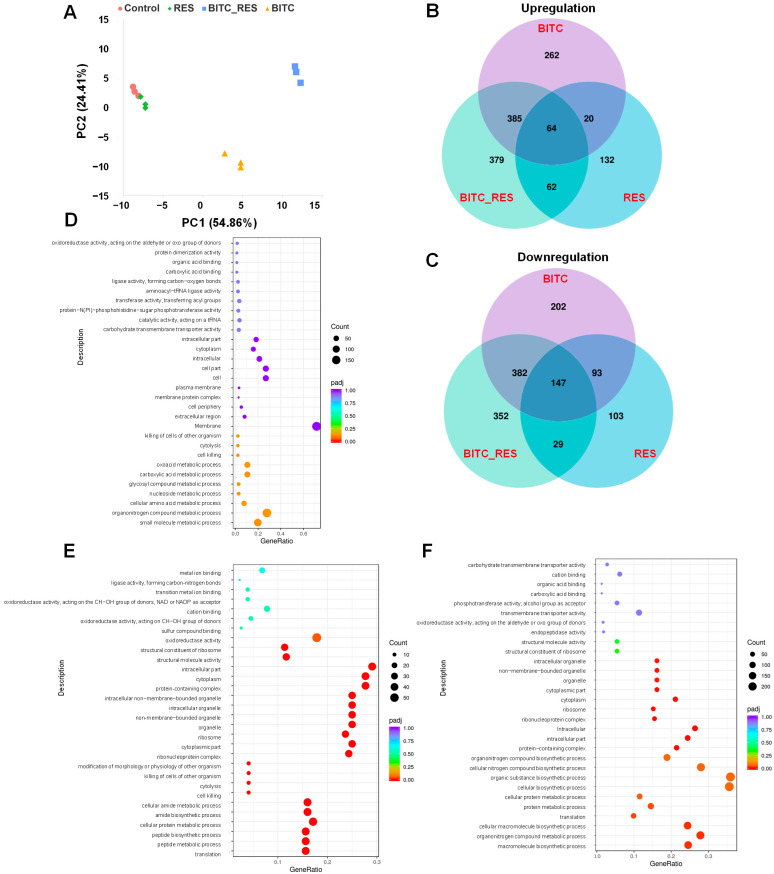
Gene expression patterns associated with the synergistic effects of BITC and RES on *S. aureus*. (**A**) PCA graph of gene expression. (**B**) Venn diagram of the number of differentially expressed genes upregulated. (**C**) Venn diagram of the number of differentially expressed genes down-regulated. (**D**) GO classification of differentially expressed genes after BITC treatment. (**E**) GO classification of differentially expressed genes after RES treatment. (**F**) GO classification of differentially expressed genes after BITC_RES treatment. *p* ≤ 0.05.

To avoid affecting cell viability, 1/8 MIC BITC (BITC), 1/8 MIC RES (RES), and 1/8 MIC BITC + 1/8 MIC RES (BITC_RES) were selected for RNA-Seq. The PCA results showed that the BITC group and the BITC_RES group induced drastic changes in gene expressions compared to the control group. Interestingly, the four groups presented different gene expression patterns (Figure 3A). In addition, Figure 3B,C shows the Venn plots of DEGs in the control and treated groups. The numbers of upregulated genes in the BITC, RES, and BITC_RES treatment groups were 731, 278, and 890, respectively. Similarly, the number of downregulated genes in the BITC_RES group was 910, which was 86 and 538 greater than those in the BITC group and RES group, respectively. Therefore, the BITC_RES group presented the greatest number of DEGs, followed by the BITC group. It has also been shown that, based on transcriptomics, resveratrol reduced the production of α-hemolysin in *S. aureus* by downregulating *saeR/S*, thereby achieving bacteriostatic effects [38]. Synergistic effects on differentially expressed genes were also seen in other bacteria. Mahamad Maifiah et al. demonstrated that polymyxin B and rifampicin in combination on *P. aeruginosa* PAO1 resulted in 823 and 445 DEGs, respectively, via Venn diagrams [18]. The study also revealed that, compared with minocycline alone, minocycline and colistin had synergistic effects on some of the DEGs for *Klebsiella pneumoniae* strains [39]. A GO enrichment analysis provides a better understanding of the biological functions of DEGs. In the BITC_RES group, the downregulated genes were most significantly enriched in biological processes, such as the heterocycle biosynthetic process. The GO terms of the upregulated genes in the biological process category were related mainly to metabolic processes, cellular metabolic processes, nitrogen compound metabolic processes, ribosomes, and organelles in the cellular composition category, and ribosomal structural components and structural molecular activities in the molecular function category (Figure 3F). Interestingly, for the individual groups, the GO enrichment differed from that of the cotreatment group, yet there were also consistent pathways, such as oxidoreductase activity and the cytoplasm (Figure 3D,E). These enriched categories are common GO terms, and similar species were also enriched by the combined treatment of p-anisaldehyde and epigallocatechin gallate, as reported by Adewunmi et al. [17]. In addition, the main GO enrichment in the BITC group were the cellular amino acid metabolic process, the carboxylic acid metabolic process, and the oxoacid metabolic process. It has also been shown that BITC treatment of methicillin-resistant *S. aureus* resulted in alterations in GO categorization, including the cellular aromatic compound metabolic process, the extracellular region part, and the nucleic acid-binding transcription factor activity, among others [6]. Significant GO enrichment in the RES treatment group included translation, peptide metabolic process, cellular protein metabolic process, structural molecule activity, and structural constituent of ribosome. Therefore, these results indicated that there were significant differences in GO enrichment of the BITC group, the RES group, and the BITC_RES group.

**Figure 4:**
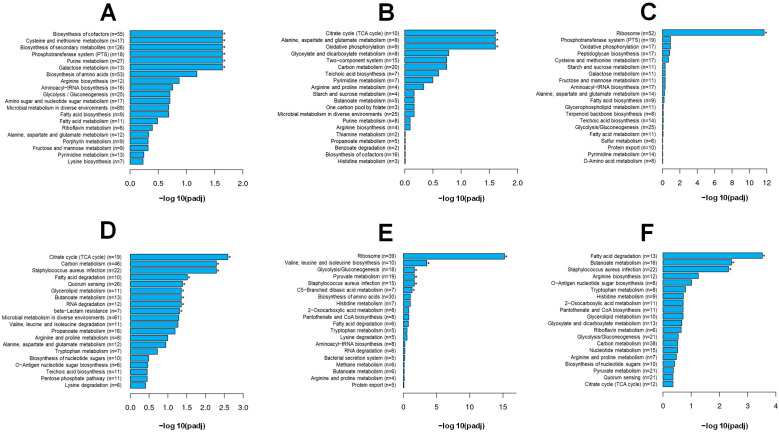
KEGG analysis of DEGs related to the synergistic effects of BITC and RES with *S. aureus*. Twenty enriched KEGG pathways are shown. * Indicates significantly enriched KEGG pathways. (**A**–**C**) Upregulated pathways; (**D**–**F**) downregulated pathways. BITC: 1/8 MIC BITC; RES: 1/8 MIC RES; BITC_RES: 1/8 MIC BITC + 1/8 MIC RES.

A KEGG pathway analysis was subsequently performed to analyze the DEGs in greater depth. Specifically, the KEGG pathways most significantly enriched in the upregulated genes in the BITC group included galactose metabolism, purine metabolism, the phosphotransferase system, biosynthesis of secondary metabolites, cysteine and methionine metabolism, and the biosynthesis of cofactors; the RES group included oxidative phosphorylation, alanine, aspartate and glutamate metabolism, and the citrate cycle (Figure 4A,B). However, the BITC_RES group included only ribosomes, suggesting that there were significant differences in the KEGG pathways between BITC and RES alone or in combination with *S. aureus* (Figure 4C). There were increased numbers of significantly downregulated pathways (Figure 4D–F). The KEGG pathways significantly enriched in the BITC_RES group were fatty acid degradation, butanoate metabolism, and *Staphylococcus aureus* infection. The BITC group was significantly enriched for more KEGG pathways, including citrate cycle, carbon metabolism, quorum sensing, RNA degradation, glycerolipid metabolism, and beta-lactam resistance, in addition to the same three pathways as the BITC_RES group. Interestingly, the *splD* gene in the quorum sensing pathway showed synergistic downregulation after BITC and RES treatment of *S. aureus*. In addition, a comparison of subinhibitory concentrations of gentamicin alone or in combination with butyrolactone I treatment of *S. aureus* KEGG showed that there was also a significant enrichment in essential biological processes, such as metabolic pathways, amino acid biosynthesis, and the ribosome [40].

### 3.5. Synergistic Effects of BITC and RES on Some Key Genes in S. aureus

**Figure 5:**
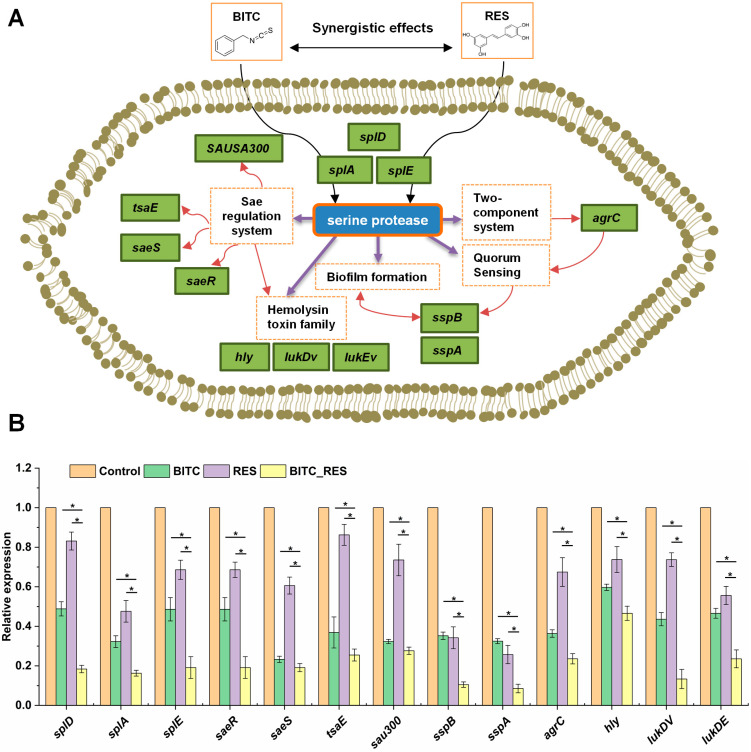
Synergistic effects of BITC and RES on some key genes in *S. aureus*. (**A**) Mechanistic diagram of *splD* regulation. (**B**) The relative expression of *splD*, *splA*, *splE*, *saeR*, *saeS*, *tsaE*, *ssau300*, *agrC*, *sspB*, *sspA*, *hly*, *lukDv*, and *lukEv* was normalized to that of the control 16S rRNA. * Gene expression differences were significant (*p* < 0.01).

*S. aureus* secretes six serine protease-like proteins (SplA-SplF) that are encoded in a single operon known as the νSaβ pathogenicity island [41]. All the Spls have a high degree of sequence homology and are particular to *S. aureus* [42]. The genes *splD*, *splA*, and *splE* are serine protease-like proteins associated with virulence [43]. The relative expression of the *splD*, *splA*, and *splE* genes decreased by 81.6%, 83.7%, and 80.9%, respectively, in the BITC_RES group, and reduced by 30.4–64.7%, 16–31.3%, 29.5–49.5% compared to the BITC group and RES group, respectively (Figure 5). The Spls are under direct regulation by the Sae regulatory system, and Sae is an extracellular protein expression site in *S. aureus* that regulates various virulence factors [44]. Histidine kinase (SaeS) and response regulator (SaeR) form a two-component system [45]. SaeR is a downstream regulator of the SaeR/S two-component system that downregulates some downstream genes, such as *sspA* and *splA* [46]. The Sae regulatory system-related genes included *saeR, saeS*, threonylcarbamoyladenosine biosynthesis protein (*tsaE*), and type VII secretion system protein (*sau300*), and the validation results of the DEGs were consistent with those of the RNA-Seq data (Table 1, Figure 5). The relative expression of the *saeR, saeS, tsaE* and *sau300* genes was reduced by 51.4–76.7% and 13.7–29.4% in the BITC group and RES group, respectively, and decreased by 4.2–29.5% and 21.5–60.8% in the BITC_RES group compared with the individual groups (Figure 5). Tao et al. also showed, via transcriptomics, that flavones downregulated the expression of the *S. aureus* genes, such as *splA, splD, splE, lukD*, *lukE, saeS*, and *saeR*, and the downregulation of some of these genes was also verified via qRT-PCR [47]. The staphylococcal proteolytic cascade is then initiated by aur, which involves the activation of the serine protease SspA (*sspA*) and the cysteine protease SspB (*sspB*) [48]. In this study, Spls regulated biofilm formation. Additionally, *sspA* and *sspB* are related to biofilm formation [49]. Quorum sensing, the intercellular communication carried out by self-induced factors, plays a crucial role in bacterial virulence [50]. *S. aureus*-specific self-inducing agents are self-inducing peptides [51]. It consists of accessory gene regulator protein A (AgrA) and accessory gene regulator protein C (AgrC). AgrC binds to the autoinducing peptide, and AgrA activates the P3 promoter and affects the transcription of RNA III. The virulence factors regulated by RNAIII are mainly *splF*, *splD*, *sspA*, etc. [52,53]. Therefore, genes that are collectively involved in biofilm formation and quorum sensing, such as *sspB, sspA*, and *agrC*, were 23.7–24.7%, 17.2–24%, and 12.7–43.8% less common in the BITC_RES group than in the BITC group and the RES group, respectively. In addition, these genes, such as *hly*, *lukDv*, and *lukEv*, had regulatory effects on the hemolysin toxin family [54]. In summary, BITC and RES showed synergy on all of the above genes (Table 1, Figure 5).

### 3.6. Synergistic Effects of BITC and RES on S. aureus in Beef at 4 °C and 25 °C

**Figure 6:**
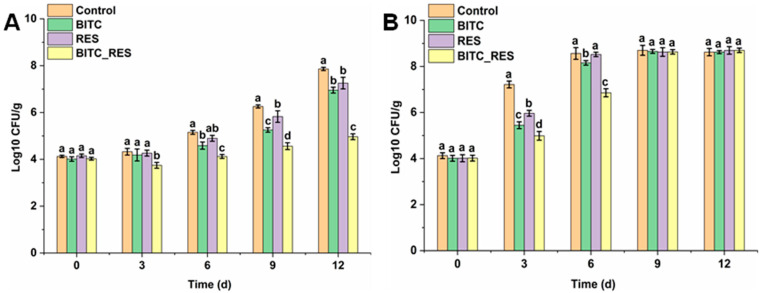
Synergistic effects of BITC and RES on *S. aureus* inoculated in lean beef: (**A**) 4 °C; (**B**) 25 °C. Different letters represent significant differences.

The ability of pathogens to remain in beef for long periods of time and proliferate rapidly poses an even greater challenge to ensuring food safety [55]. The present study has shown that the combination of BITC and RES exhibited synergistic antimicrobial activity against *S. aureus*. To further explore their synergistic effects as bacteriostatic agents in beef, lean beef was inoculated with *S. aureus* at 4 °C and 25 °C. The *S. aureus* counts in beef supplemented with BITC, RES or BITC_RES were reduced by 0.9 log CFU/g, 0.6 log CFU/g, and 2.9 log CFU/g, respectively, within 12 days at 4 °C, suggesting that the BITC combined with RES inhibited the growth of *S. aureus* in beef better than the individual groups (Figure 6A). At 25 °C, on day 6, there was no significant difference between the BITC and RES groups relative to the control group; however, the BITC_RES group significantly reduced the number of *S. aureus* by 1.7 log CFU/g (Figure 6B). After day 6, there were no significant differences among the control, BITC, RES, and BITC_RES groups (Figure 6B), which may be due to the volatility of BITC and the low bioavailability of RES, resulting in the disappearance of the antimicrobial effects of BITC and RES after 6 days [5,14]. Previous reports have shown that cell wall hydrolase Lys14579 and cinnamaldehyde synergistically inhibit *Bacillus cereus* inoculated in lean beef [19]. In addition, Endolysin LysSA97 and carvacrol also showed synergistic antibacterial activity against *S. aureus* inoculated on lean beef [20]. Therefore, the above results further indicate that BITC and RES also have synergistic antimicrobial effects against *S. aureus* in lean beef. However, the volatility of BITC, as well as certain flavors, have limitations for practical applications. Food safety requires more indicators in addition to antimicrobial activity.

## 4. Conclusions

The results of this study revealed that BITC and RES had significant synergistic antibacterial effects on *S. aureus*. BITC and RES synergistically inhibited *S. aureus* biofilm formation and disrupted cell membrane permeability. In addition, BITC and RES had synergistic effects on 13 genes related to the *splD* regulatory network according to transcriptome analysis. The molecular docking results predicted that the protein encoded by splD may be a target for BITC and RES action in *S. aureus*. Finally, BITC and RES synergistically retarded the growth of *S. aureus* in lean beef. These findings will help to explore the synergistic effects of natural products against *S. aureus*.

## Figures and Tables

**Table 1:** Effects of benzyl isothiocyanate and resveratrol on the expression of serine protease-related genes in *S. aureus*.

Gene ID	Gene Name	Protein Function	Log2 Fold Change		
			BITC	RES	BITC_RES
**Serine Protease**					
SAOUHSC_01938	*splD*	serine protease SplD	−1.5665		−8.0212
SAOUHSC_01942	*splA*	serine protease SplA	−1.4534	−0.5767	−7.3169
SAOUHSC_01936	*splE*	serine protease SplE	−0.6263		−5.8138
**Sae regulation system**					
SAOUHSC_00715	*saeR*	response regulator		0.5030	−2.1426
SAOUHSC_00714	*saeS*	sensor histidine kinase SaeS	−0.3589	0.4733	−2.0228
SAOUHSC_02280	*tsaE*	threonylcarbamoyladenosine biosynthesis protein	0.4837		−1.1128
SAOUHSC_00266	*sau* *300*	Type VII secretion system protein	−2.3866		−3.1303
**Two- component system**					
SAOUHSC_02264	*agrC*	accessory gene regulator protein C	−2.2806		−2.5308
**Quorum Sensing**					
SAOUHSC_00987	*sspB*	cysteine protease	−1.2682		−1.636
**Biofilm formation**					
SAOUHSC_00988	*sspA*	glutamyl endopeptidase	−1.1754		−1.5138
**Hemolysin toxin family**					
SAOUHSC_01121	*hly*	alpha-hemolysin	−2.5263	0.2595	−5.2095
SAOUHSC_01954	*lukDv*	leukotoxin LukD	−0.7223		−2.3529
SAOUHSC_01955	*lukEv*	leukotoxin LukE		−0.9734	−2.8215

## Data Availability

The original contributions presented in the study are included in the article/Supplementary Materials, further inquiries can be directed to the corresponding author.

## Supplementary Materials

The following supporting information can be downloaded at: https://www.mdpi.com/article/10.3390/foods14091610/s1, Table S1: Sequences of primers used in qRT-PCR.
