# Supplementary material for: Synergistic Antibacterial Mechanism of Benzyl Isothiocyanate and Resveratrol Against Staphylococcus aureus Revealed by Transcriptomic Analysis and Their Application in Beef
Source: Foods. 2025 May 1;14(9):1610. doi: 10.3390/foods14091610 (PMC12071323; doi:10.3390/foods14091610)
Supplement: Supplementary file 1 [file foods-14-01610-s001.zip › foods-3568932-supplementary.pdf]

Table S1. Sequences of primers used in qRT-PCR

Gene	Primer	Sequence (5'–3')
16S rRNA	*16S rRNA-F*	CGTGCTACAATGGACAATACA
	16S rRNA-R	ACAATCCGAACTGAGAACAAC
splD	*splD-F*	GCGGCATTGACGATTTTAAC
	splD-R	TGTATGGTGCAACATTCGTG
splA	*splA-F*	ACCGATGCAACTAAGGAACC
	splA-R	TTTCCTCCGCCTTTACCTTT
splE	*splE-F*	TGGAACAGGTTTCATTGTCG
	splE-R	ACCATTGGGATGCGCTATAA
saeR	*saeR-F*	CCAAGGGAACTCGTTTTACG
	saeR-R	ACGCATAGGGACTTCGTGAC
saeS	*saeS-F*	GCGATGAAGGTATTGGCATT
	saeS-R	TTGTTGCGCGAGTTCATTAG
tsaE	*tsaE-F*	ACTTCCAGCGACGCATTTAT
	tsaE-R	AATGAGCAGCGAATTCATGG
SAUSA300	*SAUSA300-F*	TTGCATGGTGGTCGATTGTA
	SAUSA300-R	AAAACACGCCCAATCGTATC
agrC	*agrC-F*	CCTATCATTCGCGTTGCATT
	agrC-R	CCTAAACCACGACCTTCACC
sspB	*sspB-F*	TAAAGCCAAAGCCGATTCAC
	sspB-R	CCAGCAAATTGTTGTTGTGC
sspA	*sspA-F*	CAGCAAACGCGTTATCTTCA
	sspA-R	TTGCGTGTTCACGTTGTTCT
hly	*hly-F*	GGCCTTATTGGTGCAAATGT
	hly-R	CCATATACCGGGTTCCAAGA
lukDv	*lukDv-F*	TGAATAATGGTTGGGGACCA
	lukDv-R	GCATTTGATGTGTTGGCAAG
lukEv	*lukEv-F*	GGGGTGTTAAAGCAAACGAA
	lukEv-R	GCTGAACCTGTTGGACCATT
